# Supplementary material for: Rational Use of Danofloxacin for Treatment of Mycoplasma gallisepticum in Chickens Based on the Clinical Breakpoint and Lung Microbiota Shift
Source: Antibiotics (Basel). 2022 Mar 17;11(3):403. doi: 10.3390/antibiotics11030403 (PMC8944443; doi:10.3390/antibiotics11030403)
Supplement: Supplementary file 1 [file antibiotics-11-00403-s001.zip › antibiotics-1625863-supplementary.pdf]

antibiotics

Article

Rational Use of Danofloxacin for Treatment of *Mycoplasma Gallisepticum* in Chickens Based on the Clinical Breakpoint and Lung Microbiota Shift

Shuge Wang [1,2,3], Anxiong Huang [1,2], Yufeng Gu [1,2], Jun Li [4], Lingli Huang [1,2], Xu Wang [1,2], Yanfei Tao [1,2], Zhenli Liu [1,2], Congming Wu [3], Zonghui Yuan [1,2] and Haihong Hao [1,2,*]

[1] National Reference Laboratory of Veterinary Drug Residues (HZAU) and MAO Key Laboratory for Detection of Veterinary Drug Residues, Wuhan 430070, China; shugewang@webmail.hzau.edu.cn (S.W.); anxionghuang@webmail.hzau.edu.cn (A.H.); guyufeng@webmail.hzau.edu.cn (Y.G.); huanglingli@mail.hzau.edu.cn (L.H.); wangxu@mail.hzau.edu.cn (X.W.); tyf@mail.hzau.edu.cn (Y.T.); liuzhli009@mail.hzau.edu.cn (Z.L.); yuan5802@mail.hzau.edu.cn (Z.Y.)

[2] MOA Laboratory for Risk Assessment of Quality and Safety of Livestock and Poultry Products, Wuhan 430070, China

[3] National Center for Veterinary Drug Safety Evaluation, College of Veterinary Medicine, China Agricultural University, Beijing 100193, China; wucm@cau.edu.cn

[4] Institute of Food Safety and Nutrition, Jiangsu Academy of Agricultural Sciences, Nanjing 210000, China; lijunjaas@126.com

* Correspondence: haohaihong@mail.hzau.edu.cn; Tel.: +0086-27-87287186; Fax: 0086-27-87672232

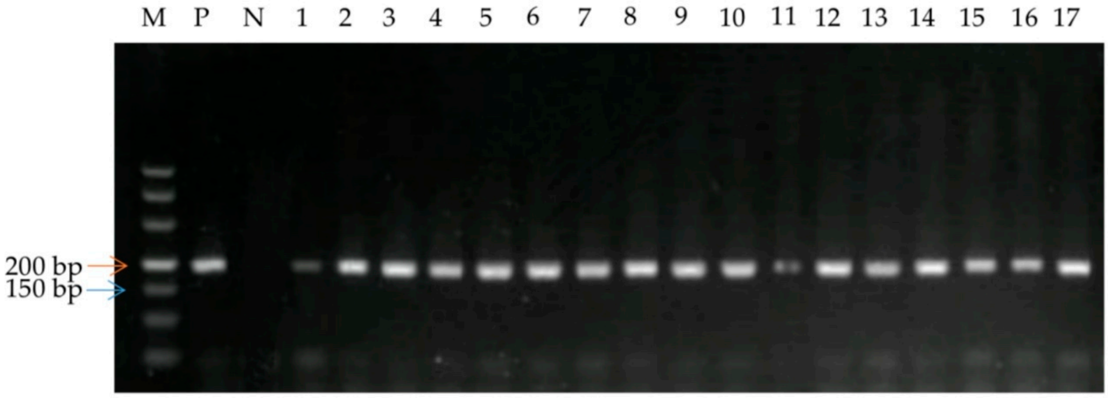

Figure S1. Electropherogram of PCR products of *MG*

Note: M is DL1000 DNA Marker， the band from the top to the bottom is 500 bp, 400 bp, 300 bp, 200 bp, 150 bp, 100 bp, 50 bp; “P” was positive control; “N” was negative control, “1-17”were samples.

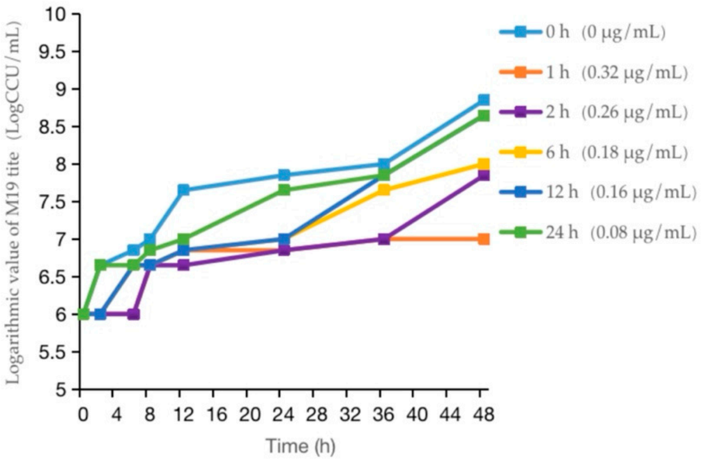

Figure S2. *Ex vivo* curves of danofloxacin against M19 in the healthy plasma.

Note: The value in brackets is the drug concentration measured by HPLC at the corresponding sampling time point.

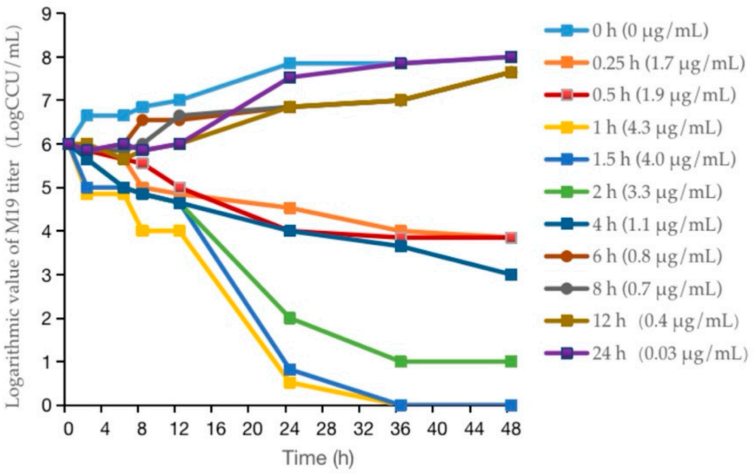

Figure S3. *Ex vivo* killing-time curves of danofloxacin against M19 in healthy lung tissue.

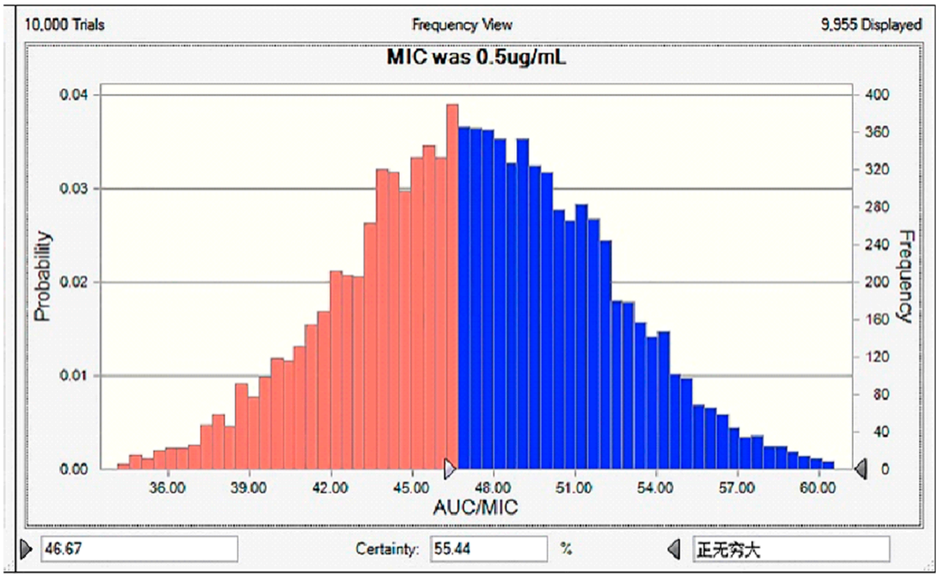

Figure S4. Probability of danofloxacin against *MG* in lung at 0.5 μg/mL

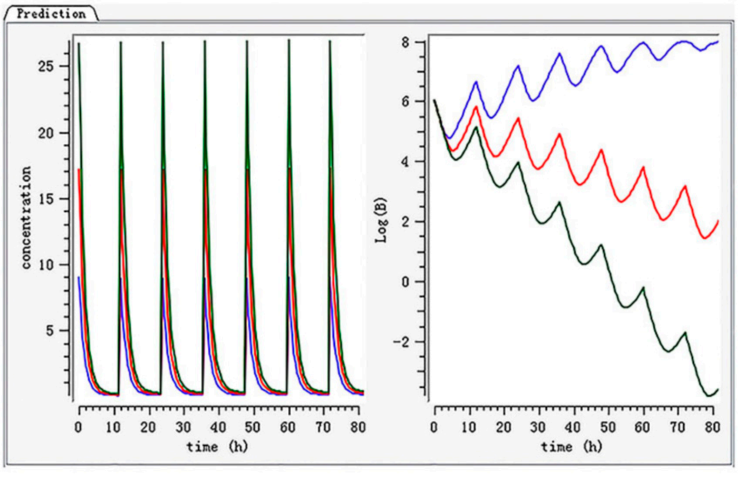

B

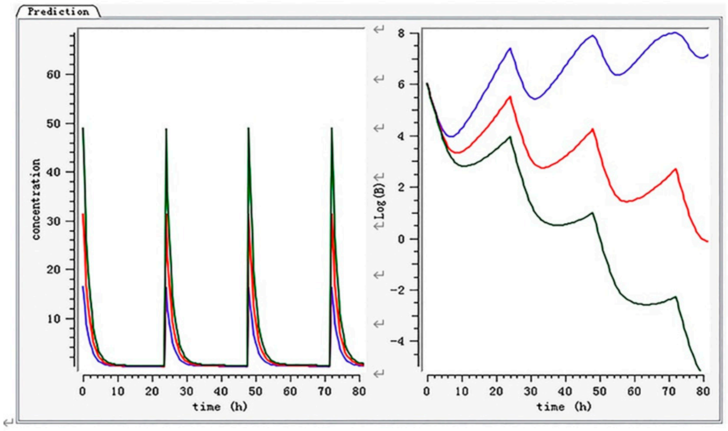

Figure S5. The growth of bacteria under different drug regiment by Mlxplore simulation

Note: Figure S5A: Blue lines represents prevent dosage (4.46 mg/kg twice a day); Red lines represents therapeutic dosage (8.80 mg/kg twice a day); Green lines represents eradicate dosage (10.37 mg/kg twice a day); Figure S5B: Blue lines represents prevent dosage (8.93 mg/kg once a day); Red lines represents therapeutic dosage (16.60 mg/kg once a day); Green lines represents eradicate dosage (20.74 mg/kg once a day).

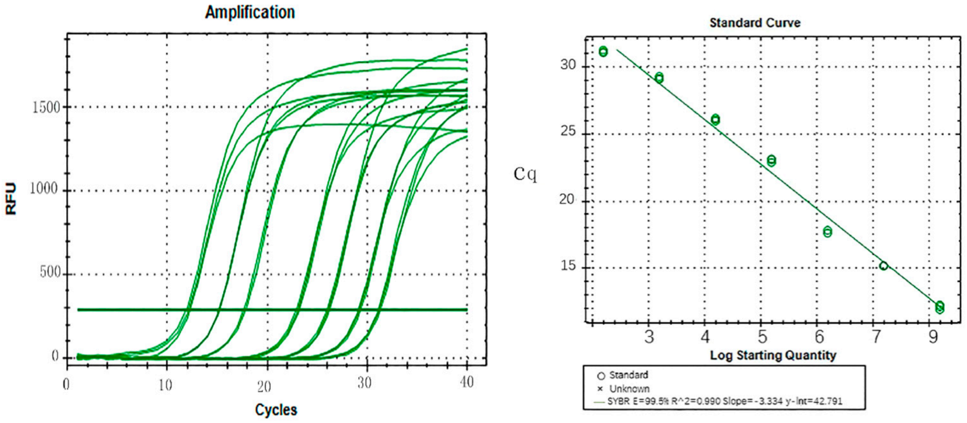

Figure S6. Dynamic curve and standard curve of recombinant plasmid (Left: amplification curve, Right: standard curve).

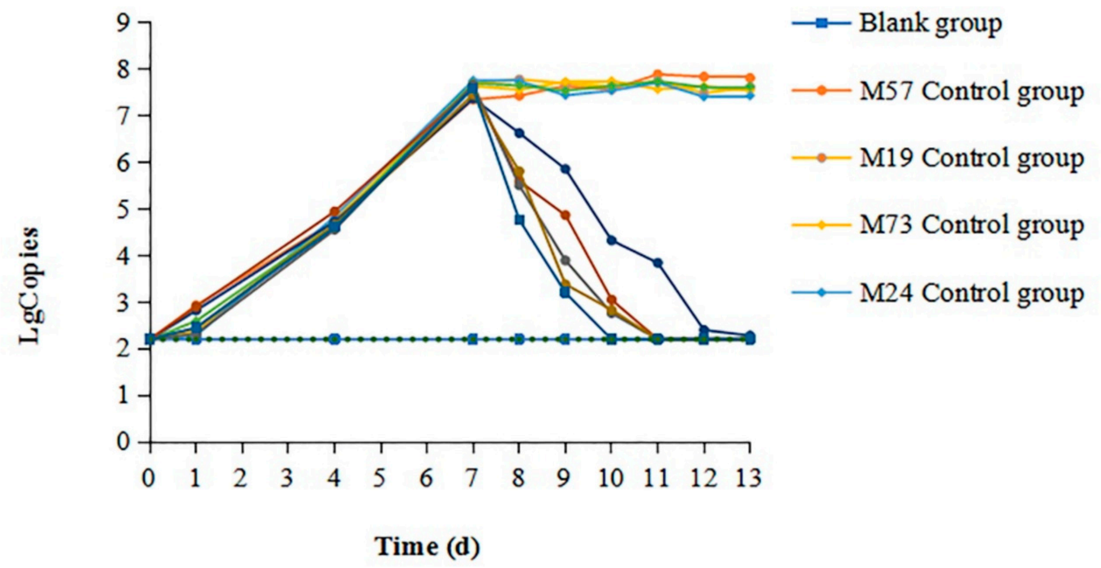

Figure S7. Logarithmic change of *MG* copy number during the test.

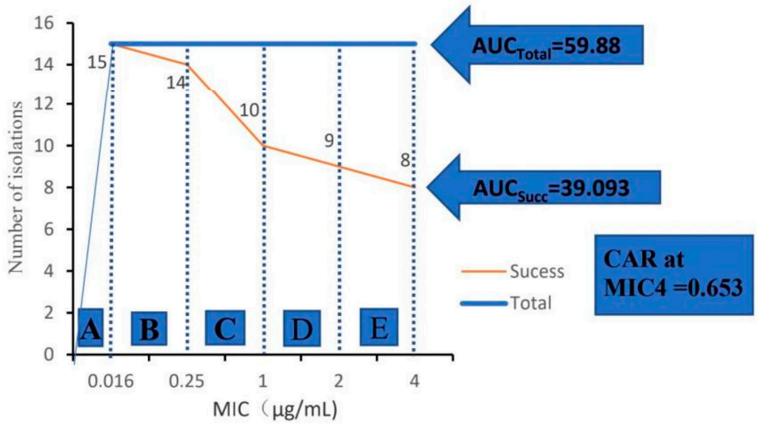

Figure S8. Graphic illustration of the CAR method.

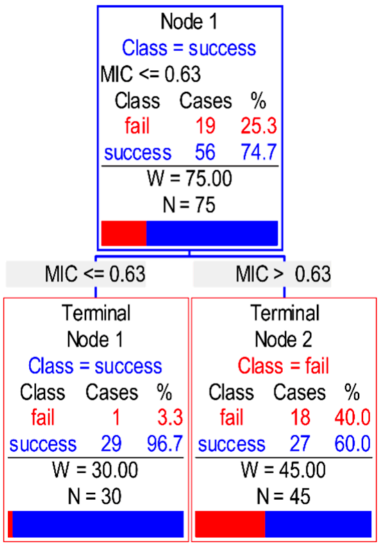

Figure S9. The results of the CART model.

Note: CART uses the Gini coefficient minimization criterion to automatically select nodes to segment the MIC and generate a regression tree. The selected node was MIC=0.63 μg/mL.

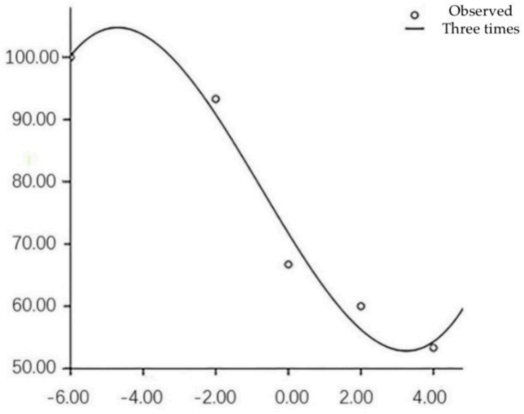

Figure S10. Non-linear regression simulation of Log_2MIC and POC

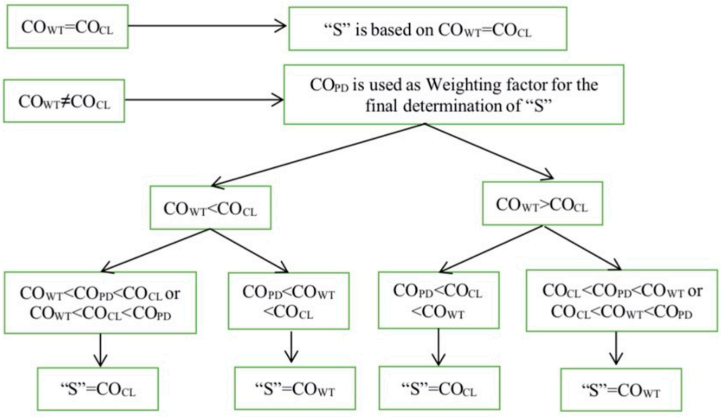

Figure S11. Susceptibility breakpoint decision tree

Table S1. The virulence test results of *MG* in embryo

Number	Death/Total	Positive/Total	Infection Rate	Death Rate
M3	3/10	10/10	100%	30%
M19	10/10	10/10	100%	100%
M29	6/10	10/10	100%	60%
M38	4/10	10/10	100%	40%
M41	1/10	10/10	100%	10%
M58	3/10	10/10	100%	30%
M77	5/10	10/10	100%	50%
M83	2/10	10/10	100%	20%
M98	8/10	10/10	100%	80%
M100	5/10	10/10	100%	50%
M107	4/10	10/10	100%	40%

Table S2. Results of Ecoffinder simulation for danofloxacin against *MG*

Parameter	Fitted Value
MIC range	0.008-8 μg/mL
MIC_{50}	1 μg/mL
MIC_{90}	2 μg/mL
Selected Subset	≦4 μg/mL
Modal MIC	0.25
Log_2MIC Mode	-2
Max Log_2MIC	3
Selected Log_2Mean	-2
Selected Log_2SD	1
95.0% Subset ECOFFs	1 μg/mL
97.5% Subset ECOFFs	1 μg/mL
99.0% Subset ECOFFs	2 μg/mL
99.5% Subset ECOFFs	2 μg/mL
99.9% Subset ECOFFs	4 μg/mL

Note: Selected Subset was the optimal fitting range by nonlinear regression; and Modal MIC was the highest MIC distribution.

Table S3. Drug concentration in chicken plasma and lung tissues at different time points after administration of danofloxacin(5 mg/kg) (n = 5)

Sampling Time (h)	Drug Concentration (μg/mL)			
	Plasma		Lung Tissue	
	Healthy Group	Diseased Group	Healthy Group	Diseased Group
0	0	0	0	0
0.25	0.20 ± 0.03	0.16 ± 0.04	1.70 ± 0.03	1.90 ± 0.08
0.5	0.30 ± 0.03	0.18 ± 0.03	1.90 ± 0.03	2.10 ± 0.11
1	0.32 ± 0.04	0.28 ± 0.06	4.30 ± 0.30	4.70 ± 0.10
1.5	0.27 ± 0.04	0.23 ± 0.03	4.00 ± 0.30	4.30 ± 0.11
2	0.26 ± 0.04	0.19 ± 0.03	3.30 ± 0.12	3.60 ± 0.13
3	0.23 ± 0.04	0.18 ± 0.03	2.60 ± 0.22	2.90 ± 0.15
4	0.22 ± 0.02	0.16 ± 0.03	1.10 ± 0.16	1.30 ± 0.17
6	0.18 ± 0.04	0.14 ± 0.05	0.80 ± 0.06	1.10 ± 0.10
8	0.17 ± 0.03	0.13 ± 0.04	0.70 ± 0.07	0.80 ± 0.11
12	0.15 ± 0.04	0.11 ± 0.02	0.40 ± 0.07	0.40 ± 0.06
24	0.08 ± 0.03	0.09 ± 0.04	0.03 ± 0.004	0.04 ± 0.008
36	ND	ND	ND	ND
48	ND	ND	ND	ND

Note: "ND" is not detected.

Table S4. The different dosages for three level of target effects

Antibacterial Effect	Weight Dose (mg/kg b.w)	Feeding Dose (mg/kg)
Bacteriostatic (E = 0)	8.93	90
Bactericidal (E = −3)	16.60	170
Eradication (E = −4)	20.74	200

Table S5. Community metrics (Chao 1, Simpson and Shannon indices) of the four groups.

**Group**	**Richness Index**	**Diversity Indices**	
	**Chao1**	**Simpson**	**Shannon**
HWK	2322.95	0.97	7.59
GW	1552.17	0.96	6.64
ZW	1744.61	0.96	6.52
ZHW	1094.55	0.88	5.18
